# Supplementary material for: The Construction and Exploration of a Comprehensive MicroRNA Centered Regulatory Network in Foxtail Millet (Setaria italica L.)
Source: Front Plant Sci. 2022 May 6;13:848474. doi: 10.3389/fpls.2022.848474 (PMC9121102; doi:10.3389/fpls.2022.848474)
Supplement: Supplementary file 3 [file Data_Sheet_3.zip › Sit-miR172b_Seita.2G381400.1_2341_TPlot.pdf]

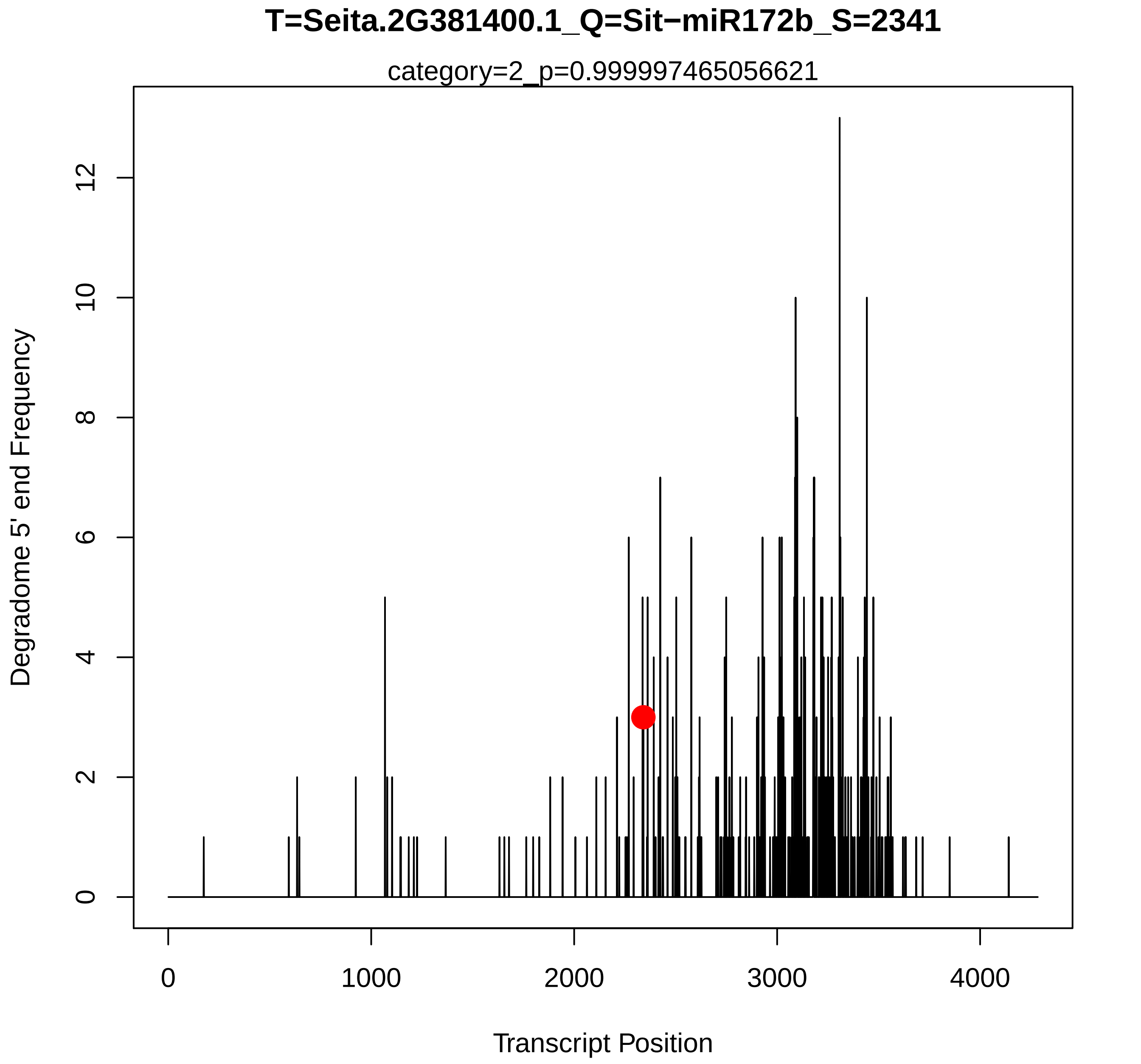
T=Seita.2G381400.1_Q=Sit−miR172b_S=2341
category=2_p=0.999997465056621
Degradome 5' end Frequency
0
2
4
6
8
10
12
0
1000
2000
3000
4000
Transcript Position
